# Evaluating Online Consumer Medication Information Systems: Comparative Online Usability Study

**DOI:** 10.2196/16648

**Published:** 2020-06-03

**Authors:** Stefan Sigle, Pilar Barriga, Francisco Javier Correa Fernández, Christian Juhra, Steffen Härtel, Christian Fegeler

**Affiliations:** 1 Department of Telemedicine University Clinic Münster University of Münster Münster Germany; 2 Center for Medical Informatics and Telemedicine Faculty of Medicine University of Chile Santiago Chile; 3 MOLIT Institute Heilbronn Germany; 4 National Center for Information Systems in Healthcare Santiago Chile; 5 Institute for Medicine, Informatics and Economy Faculty of Informatics University of Heilbronn Heilbronn Germany

**Keywords:** online consumer medication information, online usability study, sociotechnical system, information management, interoperability, implementation science

## Abstract

**Background:**

Medication is the most common intervention in health care, and the number of online consumer information systems within the pharmaceutical sector is increasing. However, online consumer information systems can be a barrier for users, imposing information asymmetries between stakeholders.

**Objective:**

The objective of this study was to quantify and compare the usability of an online consumer medication information system (OCMIS) against a reference implementation based on an interoperable information model for patients, physicians, and pharmacists.

**Methods:**

Quantitative and qualitative data were acquired from patients, physicians, and pharmacists in this online usability study. We administered 3 use cases and a post hoc questionnaire per user. Quantitative usability data including effectiveness (task success), efficiency (task time), and user satisfaction (system usability scale [SUS]) was complemented by qualitative and demographic data. Users evaluated 6 existing systems and 1 reference implementation of an OCMIS.

**Results:**

A total of 137 patients, 81 physicians, and 68 pharmacists participated in this study. Task success varied from 84% to 92% in patients, 66% to 100% in physicians, and 50% to 91% in pharmacists. Task completion time decreased over the course of the study for all but 2 OCMIS within the patient group. Due to an assumed nonnormal distribution of SUS scores, within-group comparison was done using the Kruskal-Wallis test. Patients showed differences in SUS scores (*P*=.02) and task time (*P*=.03), while physicians did not have significant differences in SUS scores (*P*=.83) and task time (*P*=.72). For pharmacists, a significant difference in SUS scores (*P*<.001) and task time (*P*=.007) was detected.

**Conclusions:**

The vendor-neutral reference implementation based on an interoperable information model was proven to be a promising approach that was not inferior to existing solutions for patients and physicians. For pharmacists, it exceeded user satisfaction scores compared to other OCMIS. This data-driven approach based on an interoperable information model enables the development of more user-tailored features to increase usability. This fosters data democratization and empowers stakeholders within the pharmaceutical sector.

## Introduction

Every medical decision is dependent on information, and thus, information quality is a key aspect when accessing health related information [1-3]. Nowadays, many people worldwide (more than 60% in Europe, 80% in the United States, and 85% of the population in low- and middle-income countries) are making use of the internet to search for information about health, medication, or medical conditions; the most frequent activity is searching for medication information [4]. This substantiates the paradigm shift toward inclusive, patient-centered health care [5] and patient empowerment [6]. Despite the fact that medication provides known benefits, adequate medication use remains a challenge for patients and providers alike [7].

Online consumer medication information systems (OCMIS) try to take on these challenges by being a source for relevant medication information among patients and providers [8]. Nevertheless, these OCMIS can also create a barrier for users that have a poor ability to read, understand, and use information to make health-related decisions; this skill is referred to as health literacy [9-11]. Moreover, the quality of information contained in a given OCMIS varies [12,13] and users may be unable to differentiate between high- and low-quality information [4]. Customized needs based on users’ preferences, skills, and knowledge are often not considered by these OCMIS [14]. This creates an information asymmetry between stakeholders, which leads to poor medication adherence, causing poorer health as well as economic issues over time [15,16].

In Chile, an emerging middle-income country in Latin America [17], the number of OCMIS within the pharmaceutical sector is increasing [18]. Within the Chilean population, 58% (and almost 90% of older citizens) take at least one type of medication, of which 88% have been indicated by a medical professional [19]. Another study reported that 30% of participants indicated that they had had to suspend a treatment because of economic reasons, which can lead to long-term health problems for citizens [20]. Governmental policies promote a rational use of medications and facilitate equal access to medications and related information through OCMIS [18,21]. However, these systems have not been evaluated for their fitness for use to date.

After a feature analysis of OCMIS as part of a systematic review [22] (Multimedia Appendix 1), this follow-up study seeks to empirically investigate the usability of OCMIS through an online usability study and simultaneously considers additional factors like health literacy.

## Methods

### Study Design

**Figure 1:**
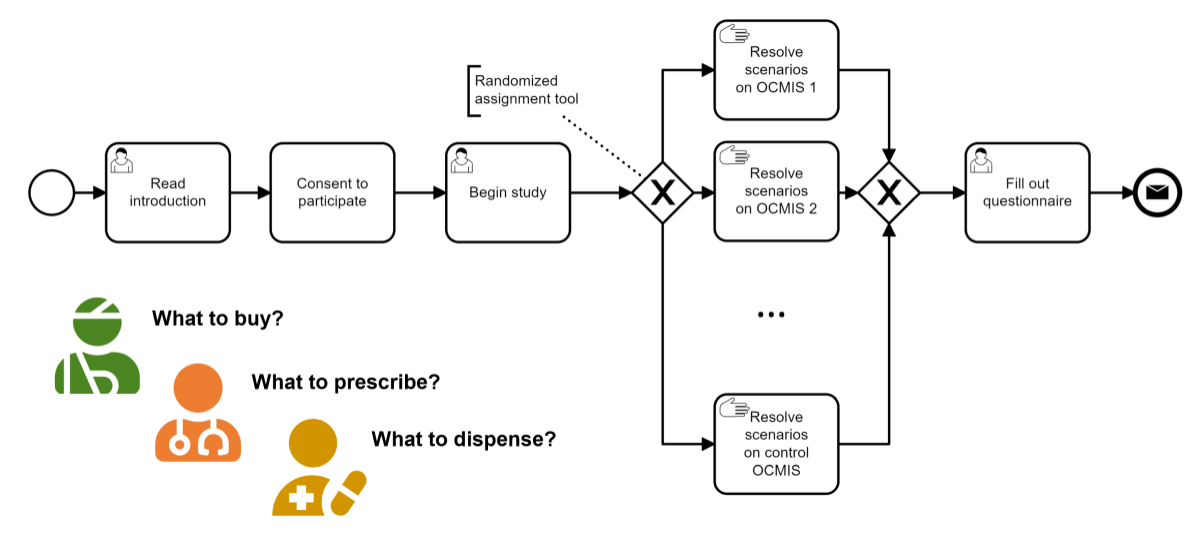
A graphical view of the study procedure is shown in a Business Process Model and Notation (BPMN). After reading the introduction and consenting to participate, the participants are randomly assigned to either the case group, which uses an online consumer medication information system (online system 1...n), or the control group, which uses the reference implementation (control system). A post hoc questionnaire was performed before concluding the study. OCMIS: online consumer medication information system.

Implementation research studies focus on real-world scenarios and identify factors that impact the uptake of research findings across multiple levels [23]. Within the context of this study, OCMIS are understood as sociotechnical systems, and the focus of this study is the human-computer interaction. This online usability study used a two-phased approach: first, there was the preceding pretest phase, which was followed by the main phase for data collection. During the pretest phase, approximately 10% of the expected participants from each group completed the study and provided feedback to researchers about the clarity and understandability of the study contents. Comments about wording obtained during the pretest were recorded as free text in digital form. Validation was performed with 2 native Spanish-speaking expert representatives from each user group and incorporated into the study after discussion. Changes to the study material were only incorporated when both experts agreed unanimously on significance and meaningfulness. Subsequently, the unmoderated main phase was conducted online, where participants acted in an in vivo setting. After the introduction video (Multimedia Appendix 2), the two-step study process was initiated: first, users completed 3 group-specific use cases with a randomly assigned OCMIS, followed by a post hoc questionnaire about the user experience during the study (Figure 1). All contents were administered in the native language. Data about the participants’ self-assessed health literacy [24,25] and OCMIS experience, as well as demographic data, were collected.

In addition, quantitative data were collected in parallel during user interaction to evaluate task success and task completion time. Data quality for the study was assured through a token system embedded in the process of accessing the study material. Pseudonymized tracking of participants without personal reference was possible, recognizing users that were not invited initially. The study was administered to participants via a URL to a self-hosted webpage where SurveyJS [26] was used for questionnaire rendering.

Participants of this study had no incentive other than to augment their knowledge about medications and OCMIS. The ethics committee at the Faculty of Medicine of the University of Chile approved this study.

### Selection of OCMIS

In discussion with 2 domain experts from each user group, 6 OCMIS were identified as relevant. For patients, domain experts were head organizers of patient interest groups. Physician experts were academic professionals with expertise in public health, and pharmacist experts were represented through academic professionals. After interacting with each of the platforms, experts selected relevant OCMIS based on the information needed to fulfill typical use cases. OCMIS were categorized as online pharmacies (Farmazon [27], Pharol [28]), web presence of a traditional pharmacy (Salcobrand [29]), government-driven (Ministry of Health [MINSAL] [30]; Public Health Institute of Chile [ISP] [31]), and supplier-driven (National Health Service System/La Central Nacional de Abastecimiento [CENABAST] [32]) OCMIS. OCMIS were assigned to user groups based on a decision matrix based on their features to ensure suitability.

**Figure 2:**
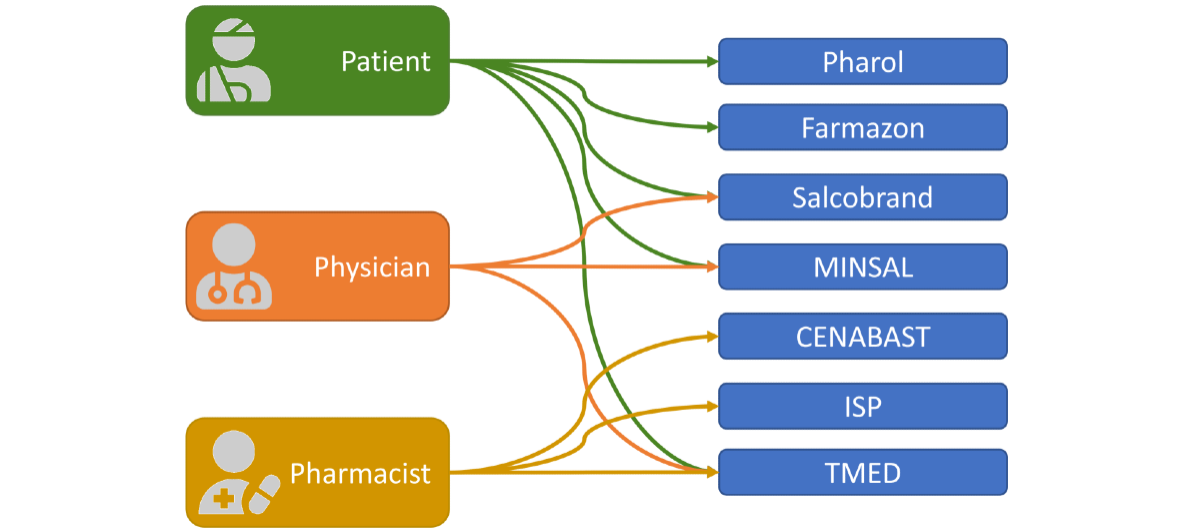
Graphical representation of the patient (green), physician (red), and pharmacist (yellow) user groups and their assigned online consumer medication information systems. Online system types include online pharmacies (Pharol, Farmazon), a traditional pharmacy (Salcobrand), medication information aggregators (MINSAL), medication information platforms (CENABAST, ISP), and a self-developed platform (TMED). CENABAST: National Health Service System of Chile; ISP: Public Health Institute of Chile; MINSAL: Ministry of Health of Chile; TMED: medication terminology.

In addition to the aforementioned OCMIS, the reference implementation TMED (medical terminology) [33], based on an interoperable information model called *Chilean Pharmaceutical Terminology* [34], was part of the test bench for all user groups (Figure 2).

TMED is the result of an effort to create the first vendor-neutral, standardized, and interoperable information database using Fast Healthcare Interoperability Resources (FHIR), a standard developed by Health Level 7. The information model accommodates the Chilean pharmaceutical sector, enabling users to search for and view bioequivalent generic and brand medications as well as innovator products [22,34,35]. TMED supports identification of medication type by qualities and features and provides the possibility of grouping medications by principal active substances. However, in order to evaluate its fitness for use, use cases had to be defined.

### Use Case Definition

Use cases are part of requirements engineering and are a narrative description of user actions and expected outcomes [36]. They allow the derivation of a feature set that must be provided by a system to the user in order to be of use. The aforementioned domain experts from each user group were prompted to write an easy-to-understand narrative text that outlines an everyday interaction with an OCMIS from their point of view, including prerequisites such as prescriptions for patients, diagnoses for physicians, and principal active substances for pharmacists (Textbox 1). Suitable exemplary medical conditions for the use cases were consented by all 6 domain experts (Textbox 2).

During the course of the study, each participant solved the use case for their group with 3 group-specific scenarios given in consecutive order, based on the use cases defined above. All scenarios are equal in structure and involve finding a medication for a specific medical condition, which facilitated participant learning and familiarization with the OCMIS. Subsequently, we established how OCMIS usability would be evaluated.

**Use case definition for the patient, physician, and pharmacist user groups.**

- Patient: finding a suitable commercial product for a prescription received from a physician.
- Physician: finding a suitable commercial product to prescribe for a patient based on a principal active substance indicated for a diagnosis.
- Pharmacist: finding a suitable commercial product to restock a pharmacy, based on the need for principal active substances issued by physicians.

**Selected medical conditions used as concrete examples for the use case.**

- Atypical pneumonia, which has a growing prevalence in the Chilean population [37].
- Focal epilepsy, one of the most common neuronal diseases worldwide; the majority of individuals with focal epilepsy (80%) live in low- and middle-income countries [38].
- Hypertension, one of the most common diseases; it affects more than 3.6 million in Chile and 1.3 billion worldwide [39].

### Evaluating System Usability

Usability evaluations are critical for assuring user acceptability when designing applications [40]. Approaches from pragmatic and academic contexts are relevant when conducting usability studies [41]. International Organization for Standardization (ISO) guideline 9241-11 includes 3 dimensions for usability: effectiveness, efficiency, and user satisfaction [42]. Effectiveness is expressed as task success, efficiency is expressed as task completion time, and user satisfaction is captured in a scoring system (eg, using the SUS).

#### Task Success

The first usability dimension was measured on 3 discrete levels: complete success, partial success, and not successful. Results were aggregated dichotomously over all 3 tasks resolved by the participant by defining anything other than a complete success as not successful. Overall success was achieved if at least 2 tasks were completed successfully by the user.

#### Task Completion Time

Task completion time in seconds was measured automatically during the study for each task and user.

#### User Satisfaction

User satisfaction was measured using the well-established SUS, which yields a score between 0 and 100 [43]. This nonproprietary, 10-item, 5-point Likert scale tool has been extensively validated and translated into multiple languages [44]. Although it is not ideal as a standalone metric, the literature suggests combining the SUS score with task success if possible [45]. The SUS itself can be broken down into 2 principal factors: usability and learnability [46]. OCMIS were rated by each participant using the SUS as a validated measure of learnability and user satisfaction [43].

### Sample Size and Internal Consistency

A sample size calculation was conducted. Literature suggests a sample size of 12-14 as sufficient to distinguish user satisfaction reliably between websites [47]. However, a sample size calculation based on a desired margin of error of 12 points in SUS score with SD of 21 and confidence level of 90%, as suggested by the literature [48], resulted in a minimum sample size of 15 participants for each platform. Internal consistency was measured using Cronbach alpha. The literature suggests acceptable values range from .70 to .90 [49,50].

### Recruitment and Data Collection

Inclusion and exclusion parameters were defined prior the study. Physicians had to have completed medical school; in Chile, this includes 2 years of practical experience in the field. Pharmacists had to have at least 1 year of professional experience. Patients were only included if they had bought medication at least once in their life. Possible participants were contacted via email invitation among special interest groups (eg, for pharmacists, invitations were sent to members of the College of Pharmaceutical and Biochemical Chemists of Chile). The data collection phase lasted 3 months and was followed up by statistical data analysis.

### Statistical Analysis

Initially, group-wise statistical tests were conducted, comparing platforms in terms of task time, task success, and SUS score. If results were statistically significant, an adjusted pairwise examination was performed to identify the significantly different feature. SUS score and task time were compared between OCMIS using the Kruskal-Wallis test for independent samples to compare means. Task success was evaluated using the chi-square test in combination with a standardized Z-score residual post hoc test. The Pearson chi-square test evaluated how likely it is that any observed difference between the sets arose by chance. Its null hypothesis states that the frequency distribution of certain events observed in a sample is consistent with a particular theoretical distribution [51]. This study evaluated the usability for OCMIS as shown in Figure 2.

## Results

### Baseline Statistics

Study participants included 136 patients, 80 physicians, and 67 pharmacists. The overall response rate was 283 of 4849 contacted individuals (5.8%). Table 1 provides an overview of study participant demographics. The mean ages across the different groups ranged from 31 to 38 years. Of the 136 patient participants, 87 (64%) were female, as were 36 of the 80 physician participants (45%) and 30 of the 68 pharmacists (45%). Self-assessed health literacy (where 5 indicated optimal health literacy and 1-4 indicated limited health literacy) of the study population varied between 30% and 35% for patients and pharmacists and peaked at over 50% within the physician group. Of the 67 pharmacists surveyed, 56 (83%) had used OCMIS prior to participating in this study, compared with 62 of 80 physicians (77%) and only 80 of 136 patients (58%). All participants from all groups reported that they used the internet on a daily basis, therefore data collected on internet use was not included in the overview.

To reduce the out-of-pocket spending for patients, 68 of 80 physicians (85%) reported considering the patient’s health insurance when prescribing medications, and 57 of 80 (71%) reported considering the economic situation of the patient.

When asked whether generics are bioequivalent to their respective innovator medication, 33 of 80 of physician participants (41%) stated that they are equal. In contrast, only 30 of 67 pharmacists surveyed (45%) agreed that innovator drugs could be replaced with generics without concern. In addition, 24 of 67 pharmacists (36%) disagreed and 12 of 67 pharmacists (18%) stated some concerns about replacing innovator drugs with generics.

**Table 1:** Baseline table of the participants.

Characteristic		Patients (n=136)	Physicians (n=80)	Pharmacists (n=67)
Age (years), mean (SD)		38 (11.2)	31 (6.2)	35 (9.2)
**Sex, n (%)**				
	Female	87 (64)	36 (45)	30 (45)
	Male	49 (36)	44 (55)	37 (55)
**Health literacy, n (%)^a^**				
	Limited	85 (65)	36 (47)	45 (68)
	Optimal	46 (35)	41 (53)	21 (32)
Professional experience, mean (SD)^b^		N/A^c^	6.57 (6.6)	8.86 (7.8)
**Previous experience with online consumer medication information systems, n (%)^d^**				
	Yes	77 (59)	60 (78)	55 (83)
	No	54 (41)	17 (22)	11 (17)
**Are generic bioequivalent medications equal to innovator medications? n (%)**				
	Yes	N/A	33 (41)	24 (36)
	No	N/A	41 (51)	30 (46)
	Other	N/A	6 (8)	12 (18)
**Observations per online consumer medication information system, n**				
	Farmazon	32	—^e^	—
	Pharol	30	—	—
	Salcobrand	44	39	—
	MINSAL^f^	15	18	—
	CENABAST^g^	—	—	20
	ISP^h^	—	—	28
	TMED^i^	15	23	19

^a^These values represent self-assessed health literacy as captured by a single-item, 5-point Likert scale where 1-4 indicated limited health literacy and 5 indicated optimal health literacy.

^b^Professional experience was measured in years since graduation from university.

^c^N/A: not applicable.

^d^If Yes was indicated, the participant had used an online consumer medication information system at least once before this study.

^e^Not applicable.

^f^MINSAL: Ministry of Health of Chile.

^g^CENABAST: National Health Service System of Chile.

^h^ISP: Public Health Institute of Chile.

^i^TMED: medication terminology.

### Task Success

**Figure 3:**
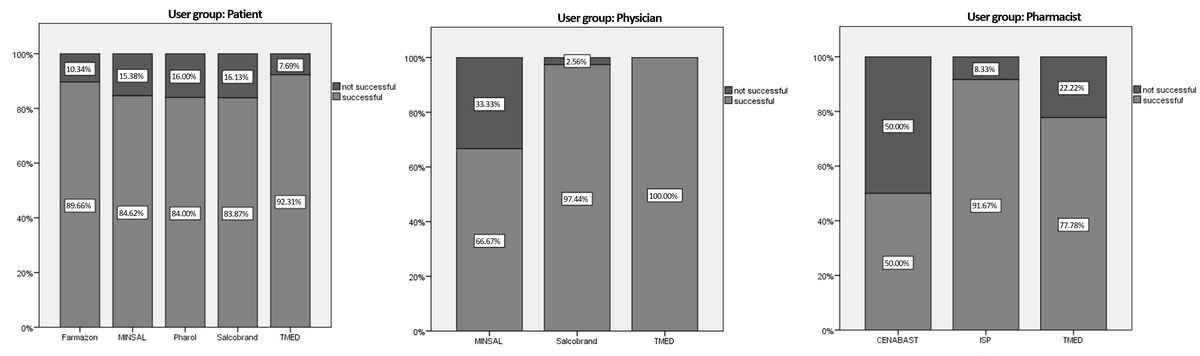
Binary task success rates for online consumer medication information systems: successful (light) and not successful (dark). CENABAST: National Health Service System of Chile; ISP: Public Health Institute of Chile; MINSAL: Ministry of Health of Chile; TMED: medication terminology.

The second usability measure was task success (Figure 3). Patients’ task success levels were relatively consistent, independent of which OCMIS was used, ranging from 84% (Pharol) to 92% (TMED).

On the other hand, physicians’ success was heavily platform-dependent, reaching a completion rate of just 67% on MINSAL and a 100% task success rate using TMED. Pharmacists’ task success rates ranged from 50% on the CENABAST platform to 92% on the ISP platform. TMED performance was in the middle of the group, with 75% of participants successfully completing the tasks.

### Task Completion Time

**Figure 4:**
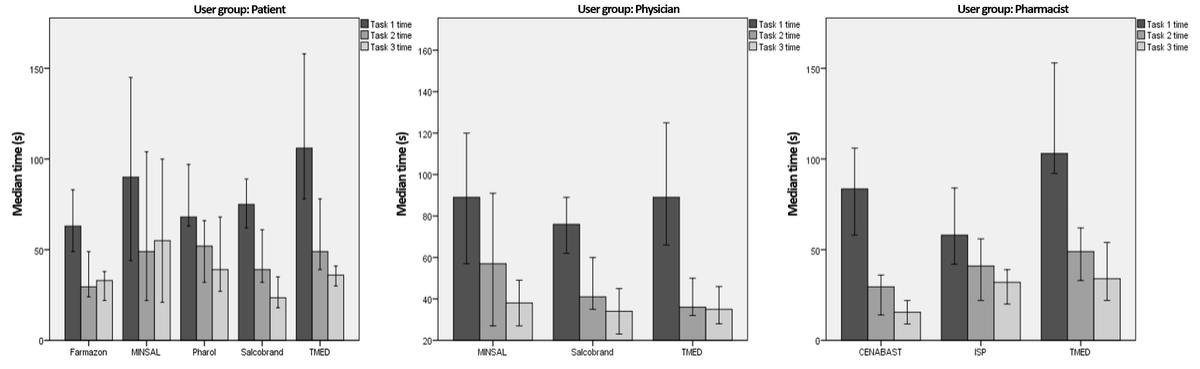
Median task completion times for patients (left), physicians (center), and pharmacists (right). Times per task 1 (dark), task 2 (lighter), and task 3 (lightest) are shown with a 95% CI. CENABAST: National Health Service System of Chile; ISP: Public Health Institute of Chile; MINSAL: Ministry of Health of Chile; TMED: medication terminology.

Median task completion time in seconds for each task is shown in Figure 4. As the 3 tasks had the same structure, we hypothesized that task times would follow a downward trend; this was confirmed overall, with the exception of Farmazon and MINSAL in the patient group, where completion times increased slightly for the second and third task. In the case of TMED, initial task times are higher than with the other systems but with later tasks, the task times approach those of other OCMIS. Physicians took the least amount of time to finish the given tasks. An aggregated comparison can be found in Table 2.

**Table 2:** Overview of task success, task time, and system usability scale scores for all user groups by online consumer medication information system.

Characteristic		Farmazon (n_Pat_^a^=32)	Pharol (n_Pat_=30)	Salcobrand (n_Pat_=44, n_Phy_^b^=39)	MINSAL^c^ (n_Pat_=15, n_Phy_=18)	CENABAST^d^ (n_Pha_^e^ =20)	ISP^f^ (n_Pha_ =28)	TMED^g^ (n_Pat_=15, n_Phy_=23, n_Pha_=19)
**Task success rate^h^, n (%)**								
	Patient	89.6	84.0	83.8	84.6	—^i^	—	92.3
	Physician	—	—	97.4	66.7	—	—	100
	Pharmacist	—	—	—	—	50.0	91.7	77.8
**Median task time^j^, n (SD)**								
	Patient	50.33 (27.61)	60.67 (50.53)	51.33 (74.03)	63.68 (61.89)	—	—	64.33 (32.65)
	Physician	—	—	50.00 (236.52)	61.00 (478.19)	—	—	56.67 (179.78)
	Pharmacist	—	—	—	—	42.33 (42.55)	47.67 (31.54)	68.00 (33.03)
**Mean SUS score^k^, n (SD, 95% CI)**								
	Patient	83.83 (15.18, 78.46-89.74)	76.38 (19.71, 69.13-84.11)	66.73 (23.87, 59.52-74.39)	71.33 (24.72, 58.02-85.31)	—	—	72.67 (15.36, 64.41-81.32)
	Physician	—	—	79.66 (15.89, 74.61-85.22)	77.06 (22.45, 65.69-88.78)	—	—	76.85 (17.23, 69.66-84.60)
	Pharmacist	—	—	—	—	50.63 (22.24, 40.43-61.27)	79.81 (20.68, 71.87-88.21)	84.87 (11.62, 79.50-90.71)

^a^n_Pat_: number of patients.

^b^n_Phy_: number of physicians.

^c^MINSAL: Ministry of Health of Chile.

^d^CENABAST: National Health Service System of Chile.

^e^n_Pha_: number of pharmacists.

^f^ISP: Public Health Institute of Chile.

^g^TMED: medication terminology.

^h^Percentage of aggregated task success rates.

^i^Not applicable.

^j^The median task time is in seconds.

^k^SUS: system usability scale; scores can be values between 0 and 100.

### User Satisfaction

**Figure 5:**
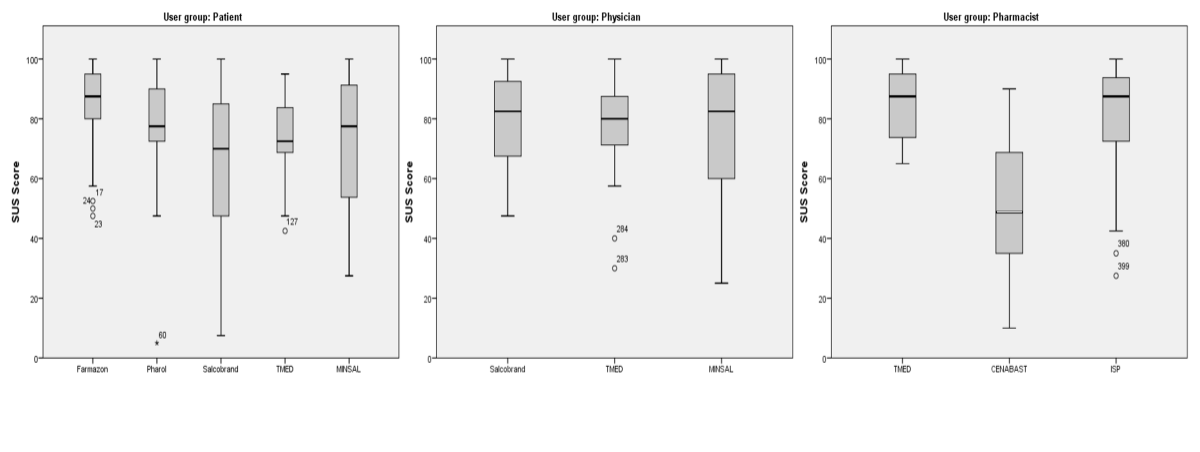
SUS box plots: the lower (Q1) and upper (Q3) quartile, representing observations outside the 9-91 percentile range. The diagram also shows the median observation. Data falling outside the Q1-Q3 range are plotted as outliers. CENABAST: National Health Service System of Chile; ISP: Public Health Institute of Chile; MINSAL: Ministry of Health of Chile; SUS: system usability scale; TMED: medication terminology.

The third dimension of usability, user satisfaction, proved to have a very high overall internal consistency, as indicated by a Cronbach alpha value of .89 for SUS scores. With one exception each in the patient and pharmacist groups, median SUS scores were above the global average of 68 (SD 12.5) for SUS scores for websites (Figure 5) [40].

#### Group-Wise Comparison for TMED

The observed mean SUS scores for TMED ranged from 72.5 (SD 15.36) for patients to 76.85 (SD 17.23) for physicians and 84.87 (SD 11.62) for pharmacists (Table 3). SUS scores among physicians and pharmacists indicate a potential for them to be net promoters of the platform. The SUS scores were transformed into percentiles [48], adjectives, and grades [52,53] to facilitate interpretation and groupwise comparison (Table 3).

**Table 3:** Transformation of TMED system usability scale scores into percentile ranks, adjectives, and grades for patients, physicians, and pharmacists.

Parameters	Patients	Physicians	Pharmacists
System usability scale score, mean (SD)	72.67 (15.36)	76.85 (17.23)	84.87 (11.62)
Percentage	66.9	88.0	96.6
Adjective	Good	Excellent	Excellent
Grade (Bangor [52])	C	B	B
Grade (Sauro & Lewis [53])	B–	A–	A+

### Statistical Evaluation

The null hypothesis was defined as not exhibiting any differences for any of the given aspects (task time, task success, SUS score), with α=.05. Due to data skewness, normality was not assumed and subsequently only nonparametrical tests were performed.

#### Patients

In the patient group, the differences in SUS scores (*P*=.02) and task time (*P*=.03) across OCMIS were significant, such that the null hypothesis was rejected. Pairwise SUS score comparison revealed an adjusted significant difference for Salcobrand and Farmazon (*P*=.008). In addition, Farmazon and Pharol differed significantly (*P*=.06) in pairwise completion times. However, task success did not differ significantly from expected values (*P*=.91).

#### Physicians

For the physician group, the differences in SUS scores (*P*=.08) and task time (*P*=.72) did not reach significant levels. No consecutive pairwise comparison was conducted. However, the differences in task success proved significant (*P*<.001) under the chi-square test. After a within-group adjustment (α=.008), MINSAL was identified as deviating significantly (*P*<.001).

#### Pharmacists

The results from the pharmacist group indicated a highly significant difference between OCMIS for SUS scores (*P*<.001) and task completion times (*P*=.007). An adjusted pairwise comparison for SUS scores revealed a significant difference between CENABAST (*P*<.001) and ISP as well as CENABAST and TMED (*P<*.001). When focusing on completion time, only CENABAST and TMED showed significant differences (*P*<.005). The differences in task success among pharmacists was significant (*P*=.008); after a post hoc adjustment (α=.008), the CENABAST OCMIS was found to deviate from expected values (*P*=.004).

### Qualitative Data

In addition to quantitative data, 76 of 136 patients (55%), 36 of 80 physicians (45%), and 31 of 67 pharmacists (46%) provided qualitative feedback about features that they considered desirable for OCMIS. Comments were analyzed for their content and tagged by keyword (Textbox 3).

**User comments on critical features for online consumer medication information systems, ranked by overall occurrence.**

- The up-to-date or approximated medication price should be displayed (132 mentions).
- Search flexibility should be increased (eg, searching for principal active substances or quality parameters; 11 mentions).
- Disambiguation of search terms (eg, phonetic searches) should be provided (10 mentions).
- Medication concentrations should be displayed (6 mentions).
- Adverse effect information should be provided (6 mentions).
- An increased amount of information about medications (eg, kinetics and posology) should be included (4 mentions).
- Evidence for medications should be shown (3 mentions).
- Filters for information such as dosage or concentration should be implemented (3 mentions).
- Integration to other knowledge databases should be considered (3 mentions).
- Georeferenced information for pharmacies and stock considerations should be included (2 mentions).
- Personal discounts due to insurance coverage should be included in the price calculation (1 mention).
- Information neutrality should be a priority (1 mention).
- Native mobile applications should be preferred (1 mention).

## Discussion

### Principal Findings

An online usability study was conducted to evaluate OCMIS on the dimensions of task success (completion), task completion time, and user satisfaction.

The ongoing controversy of whether to prescribe innovator medications or use bioequivalent generic products is reflected within the study population. Generally, physicians are slightly more confident in using generic products than pharmacists.

For patients, online pharmacies (Farmazon and Pharol) seemed to be the most suited to their tasks as indicated by high user satisfaction scores. Task time was significantly lower for the OCMIS of traditional pharmacies when compared to online pharmacies. Task success rates indicated that all platforms seemed to be suited for the use case.

Physicians seemed to have difficulties completing their tasks when using the MINSAL platform, but not when using the OCMIS of traditional pharmacies (Salcobrand) or the reference implementation (TMED).

The user satisfaction scores of pharmacists identified both ISP and TMED as the most usable platforms, with no significant difference in user satisfaction between them. The platform of public medication supplier CENABAST received lower SUS scores and also had lower task success rates.

### Strengths and Limitations

For the selection of OCMIS, a discussion with 2 professional representatives was conducted; this may not be representative of which OCMIS are used by health care professionals on a national level. However, more than half of the participants indicated an awareness of the OCMIS presented in this study, indicating that the selected OCMIS were relevant. Health literacy was not homogeneous among participants, indicating unequal starting conditions for each participant; however, this reflects reality. Participant recruitment was carried out by email distribution to special interest groups, which might introduce bias as these individuals may have a higher awareness of OCMIS.

Due to the design of online usability studies, a unique combination of advantages was achieved. The study was not moderated and no social desirability response bias [54] was introduced by this in vivo setting, assuring the most natural conditions for the user while they evaluated the OCMIS. The study design facilitated the automated collection of qualitative and quantitative data directly after the experience. In comparison to traditional usability studies, a higher number of participants was recruited in a shorter time frame, which contributed to the robustness of the results.

### Conclusions

This study demonstrated that TMED is a promising approach and showed that interoperable, neutral information models can empower stakeholders in context-agnostic medication decisions. Although an independent group should verify these results to avoid any potential bias, TMED was statistically proven to not be inferior to other OCMIS in usability aspects, while offering flexible search and extension capabilities due to its underlying interoperable information model.

Based on the results and qualitative feedback on desired features provided by participants, improvements can be incorporated to alleviate information asymmetries and foster data democratization within the pharmaceutical sector even further by providing user-tailored information. The approach of personalized drug information provision is promising and can serve as a basis for other applications, such as electronic prescriptions, and enable research opportunities through its standardized approach.

## Appendix

Multimedia Appendix 1 Benchmarking of online consumer medication information systems. This benchmark was used in a previous publication [22].

Multimedia Appendix 2 Introduction and overview video for the online usability study.

## Abbreviations

CENABAST: National Health Service System/La Central Nacional de Abastecimiento
FHIR: Fast Healthcare Interoperability Resources
ISP: Public Health Institute of Chile
MINSAL: Ministry of Health
OCMIS: online consumer medication information system(s)
SUS: system usability scale
TMED: medication terminology (Spanish: Terminologia de Medicamentos)
