# Supplementary figures and images for: Evaluating Online Consumer Medication Information Systems: Comparative Online Usability Study
Source: JMIR Mhealth Uhealth. 2020 Jun 3;8(6):e16648. doi: 10.2196/16648 (PMC7301258; doi:10.2196/16648)

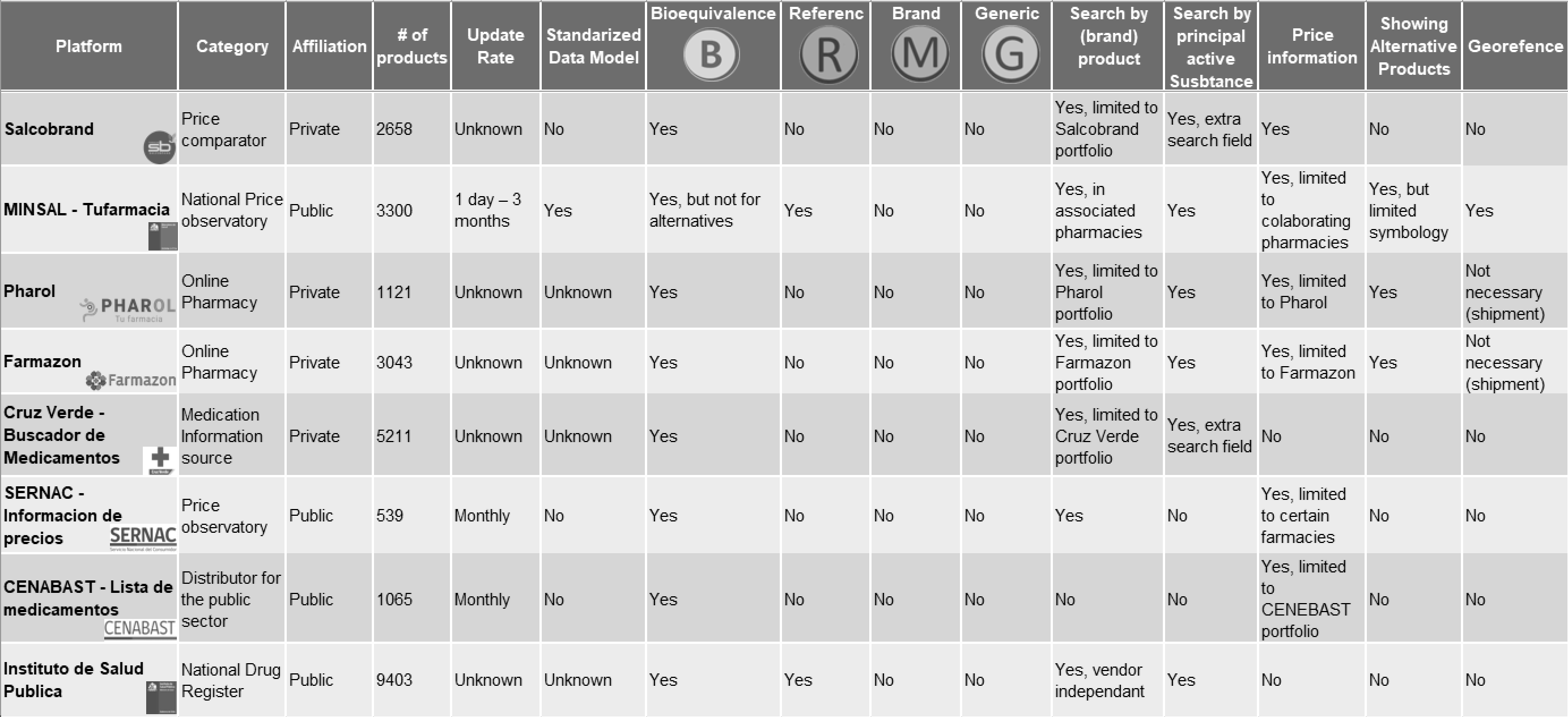

Supplement: Multimedia Appendix 1 [file mhealth_v8i6e16648_app1.png]
